# Profile of bile acid subspecies is similar in blood and follicular fluid of cattle

**DOI:** 10.1002/vms3.217

**Published:** 2019-11-11

**Authors:** Carina Blaschka, Alberto Sánchez‐Guijo, Stefan A. Wudy, Christine Wrenzycki

**Affiliations:** ^1^ Clinic for Veterinary Obstetrics, Gynecology and Andrology Molecular Reproductive Medicine Justus‐Liebig‐University Giessen Germany; ^2^ Steroid Research and Mass Spectrometry Unit Center of Child and Adolescent Medicine Faculty of Medicine Justus‐Liebig‐University Giessen Germany; ^3^ Present address: Livestock Biotechnology and Reproduction Faculty of Agricultural Sciences Georg‐August‐University Goettingen Germany

**Keywords:** Body fluids, Bovine, Cholic acid, Glycocholic acid

## Abstract

The composition of follicular fluid (FF) has an impact on the developmental capacity of the oocyte and the resulting embryo. FF is composed of blood plasma constituents which cross the blood follicular barrier and the secretory components of granulosa and theca cells. Moreover, it has been shown recently that follicular cells have the ability to synthesize bile acids (BAs). BAs are present in several fluids of mammals especially in bile, blood and urine. FF is an essential impacting factor on the oocyte quality and therefore resulting embryos. To achieve a better understanding of this subject, the presence and concentration of BAs were measured in fluid collected from bovine follicles, categorized according to their size, throughout two entire oestrus cycles and compared to those in blood and urine. The body fluids were collected during the same examination procedure and in total samples from four heifers were obtained. A broad spectrum of 11 BA derivatives was measured applying liquid chromatography–tandem mass spectrometry (LC‐MS/MS). The simultaneous and direct quantification of BAs in different body fluids of cattle are reported. Within the follicular fluid, blood and urine, cholic acid and glycocholic acid are the dominant BA subspecies irrespective of the oestrus cycle stage. Moreover, BA concentrations in blood compared to those in the FF were similar. For the first time these results clearly highlight the presence of different BA subspecies in FF, blood and urine during the oestrus cycle in cattle.

## Graphical abstract

Bile acids have been identified recently in follicular fluid of humans and cattle. To achieve a better understanding of bile acids during reproduction, the presence and concentration of bile acids were measured in fluid collected from bovine follicles throughout the oestrus cycle and compared to those in blood and urine.

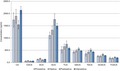

## 1 INTRODUCTION

Bile acids (BAs) are well known as main functional components of the bile. Usually, bile is secreted by hepatocytes and is stored and concentrated in the gallbladder. During digestion, bile is secreted into the lumen of the small intestine, where its acids act as emulsifiers of nutritional components (Russell, 2003). From the intestine, BAs are absorbed, re‐transported to the liver via portal circulation and then re‐secreted into the bile (Arias et al., 2009). BAs can also be cytotoxic and exhibit pathological effects if they accumulate in high concentrations. In humans it is known that BAs can be efficiently eliminated in urine. They are mainly sulphated, which increase the solubility and decrease the intestinal resorption, for urinary excretion (Alnouti, 2009).

BAs, particular primary BAs, are synthesized from cholesterol by the liver (Russell, 2003). Cholic and chenodeoxycholic acids (CA; CDCA) are the primary BAs in most species of domestic animals (Elliott & Hyde, 1971). Microorganisms in the small intestine transform these into secondary BAs, like deoxycholic and lithocholic acids (DCA; LCA; (Lefebvre, Cariou, Lien, Kuipers, & Staels, 2009)). In addition, primary and secondary BAs can be conjugated with amino acids, either glycine or taurine, which are then designated as bile salts (Elliott & Hyde, 1971). Conjugated BAs are: glycocholic acid (GCA), taurocholic acid (TCA), glycodeoxycholic acid (GDCA), taurodeoxycholic acid (TDCA), glycochenodeoxycholic acid (GCDCA), taurochenodeoxycholic acid (TCDCA), glycolithocholic acid (GLCA) and taurolithocholic acid (TLCA).

BAs are present in several body fluids like bile, blood and urine. Recently, BAs were also detected in follicular fluid (FF) of humans (Nagy et al., 2015; Smith et al., 2009) and cattle (Sanchez et al., 2014; Sanchez‐Guijo, Blaschka, Hartmann, Wrenzycki, & Wudy, 2016). Smith et al. (2009) determined the total BA concentration in human FF depending on an hCG (human chorionic gonadotropin) treatment. They detected a significant higher concentration of BAs in FF compared to serum and postulated BA production via follicular cells. In humans, Nagy et al. (2015) also determined a significant higher concentration of BAs in the fluid of follicles than in serum. Moreover, they associated individual follicular BA subspecies with embryo quality. Sanchez et al. (2014) compared total BA concentration in plasma and FF of lactating cows and heifers. Plasma and FF of lactating cows contained a significant higher concentration of total BAs than that of heifers. Recently, different BA subspecies have been quantified in FF derived from follicles of different sizes (3–5 mm, 6–8 mm, 9–14 mm, ≥15 mm) collected from abattoir‐derived ovaries during interoestrus. The primary BA CA and the corresponding conjugated form GCA showed the highest concentrations irrespective of the follicle size (Sanchez‐Guijo, Blaschka, et al., 2016).

FF is composed of blood plasma constituents, which cross the blood follicular barrier and the secretory components of granulosa and theca cells (Fortune, 1994; Hennet & Combelles, 2012). Thus the FF consists of a large amount of components such as proteins, cytokines, growth factors, peptide hormones, steroids, energy metabolites and other unknown components (Leroy et al., 2011; Sutton, Gilchrist, & Thompson, 2003; Van Hoeck et al., 2013; Wrenzycki & Stinshoff, 2013). Therefore, the composition of FF has an impact on the developmental capacity of the oocyte and the resulting embryo. But the function of BA in FF and their role within folliculogenesis is still unknown. Moreover, the origin of the BAs is largely unclarified.

The increase in knowledge related to the pattern of the BA subspecies depending on the oestrus cycle stage in cattle will provide a better understanding of the physiological processes and their possible relevance in fertility. Moreover, the extended knowledge may be used for the improvement in in vitro production systems of oocytes and pre‐implantation embryos.

Therefore, the aim of the present study was to quantify and compare the BA subspecies in FF collected at a defined oestrus cycle stage and in blood and urine samples obtained throughout the entire oestrus cycle of heifers.

## 2 MATERIAL AND METHODS

### 2.1 Collection of follicular fluid via ovum pick‐up (OPU)

Follicular fluid was collected from four heifers provided by Masterrind GmbH (ET station, Loxstedt, Germany). Undisturbed general physical conditions as well as sound gynaecological health were required. Before the individual OPU sessions, the animals received an epidural anaesthesia according to their respective body weight (2% procainhydrochlorid, 2% procasel, Selectavet, Weyam‐Holzolling, Germany).

All experiments with animals were conducted according the German ethical guidelines of animal welfare, which were approved by regional council (Regierungspräsidium) Giessen of the State of Hesse (Reference numbers V 54–19 c 20 15 hr 02 GI 18/14 Nr. A1/2016).

The ultrasound guided aspiration of FF was implemented with a GE Logiq e ultrasound unit combined with a 7.5 MHz‐probe (type 8C‐RS, GE Health care, Munich, Germany). The carrier for the ultrasound probe was individually developed, handmade by the precision mechanics of the Institute of Farm Animal Genetics of the Friedrich‐Loeffler‐Institute in Mariensee, Germany and equipped with a needle guide. The aspiration needle is a standard commercial cannula (20G x 2 ¾ " (0.9 x 70 mm) and a short bevel) of Terumo from Leuven, Belgium.

All visible follicles were categorized according to their size (3–5 mm, 6–8 mm, 9–14 mm; ≥15 mm). The aspiration was performed as described (Kruip, Boni, Wurth, Roelofsen, & Pieterse, 1994; Pieterse, Kappen, Kruip, & Taverne, 1988). Each ovary was transrectally fixed and placed in front of the ultrasound probe. The follicles were punctured through the vaginal wall. The aspiration was deemed successful if the cavity of the follicle collapsed during puncture. Pathological changes (intrafollicular haematoma, a slight hardening of the ovarian tunica albuginea, fibrous tissue accumulation around the ovaries) are described in the context of the OPU, but experience with the use of this method shows that no major negative effects on reproductive potential of animals are to be feared (Petyim, Bage, Forsberg, Rodriguez‐Martinez, & Larsson, 2000, 2001).

After the puncture of the different follicle classes, the aspiration needle was changed and the tube was rinsed with phosphate‐buffered saline (PBS) without bovine serum albumin. Until arrival at the laboratory, the samples were stored on ice at least for 40 min.

At the laboratory, the samples were centrifuged for 3 min at 300 *g* to separate the fluid from components of any cells and the supernatant was stored at −20°C until further analyses.

### 2.2 Collection of blood samples

Blood samples were collected during two physiological oestrus cycles (three healthy heifers) and before the OPU sessions (four different healthy heifers).

The animals were examined via rectal palpation, sonography (LOGIQ e, GE Healthcare, Solingen, Germany) and vaginoscopy to assess the oestrus stage. They were categorized according to the oestrus cycle stage based on morphological criteria (Hafez & Hafez, 2000) and following the nomenclature published by Senger (2005).

Blood was obtained from the vein at the tail (vena caudalis mediana). The samples were centrifuged (300 *g* for 5 min at room temperature), the serum was collected and stored at −20°C until further analyses.

### 2.3 Collection of urine samples

At each OPU session, urine samples were collected from each animal. The external genital tract was cleaned and massaged. The procedure took place before blood was taken and the animals got the epidural anaesthesia. The urine samples were stored at −20°C until further analyses.

### 2.4 Analyses of bile acids via liquid chromatography–tandem mass spectrometry (LC‐MS/MS)

FF, serum and urine collected within the OPU sessions and serum obtained during the oestrus cycles were analysed by LC‐MS/MS.

The sample preparation and the BA quantification followed the procedure outlined by Sanchez‐Guijo, Blaschka, et al. (2016). Biological fluid (200 µl) was incubated after the addition of deuterated internal standards with gently shaking for 30 min at room temperature. After protein precipitation, the supernatant was collected and mixed with 3 ml of water in a glass tube. After activation of SepPak C18 cartridges (Water Corporation), the content of the glass tube was added to the cartridge and washed with 3 ml of water followed by 6 ml of hexane. BAs were eluted from the cartridge with 5 ml methanol. After evaporation of the methanol under nitrogen stream, the samples were reconstituted in 250 μl of a solution containing 50% of MeOH, 49.75% of water and 0.25% ammonium hydroxide. The reconstituted samples were centrifuged and 10 µl of the supernatants was injected in the LC‐MS/MS system with measurements performed as triplicates. The analytical column was an Accucore Phenyl‐X column (50 × 4.6 mm, 2.6 μm), from Thermo Fisher Scientific (Dreieich, Germany). The HPLC system, an Agilent 1200SL, was connected to a triple quadrupole mass spectrometer (TSQ, Quantum Ultra, Thermo Fisher Scientific, Dreieich, Germany) using electrospray ionization in negative detection mode (Sanchez‐Guijo, Blaschka, et al., 2016). This method allows the simultaneous measurement of the following BAs: CA, CDCA, DCA, GCA, TCA, GDCA, TDCA, GCDCA, TCDCA, GLCA, TLCA. Limits of quantification (LOQ) were 10 ng/ml for DCA, LCA, GDCA, GCDCA, GLCA and TLCA; 15 ng/ml for TDCA and TCDCA; 20 ng/ml for CA and GCA; 25 ng/ml for CDCA and TCA.

As no differences could be detected related to the size of the follicles the fluid stems from, data were pooled.

Blood collected during the physiological oestrus cycle was also analysed for free (unesterified) cholesterol as reported by Sanchez‐Guijo, Neunzig, et al. (2016), LOQ: 60 nmol/L.

### 2.5 Analyses of estradiol‐17β and progesterone via radioimmunoassay (RIA)

The serum obtained from the animals during two oestrus cycles and the OPU sessions was also analysed via RIA. The determination of estradiol‐17β (E2) and progesterone (P4) via RIA was applied to verify retrospectively the supposed cycle stage at the day of sampling.

Concentrations of P4 and E2 were determined in duplicate using well‐established radio‐immunological methods after extraction of the samples with organic solvents. The measurement of P4 followed the procedure outlined by Hoffmann, Kyrein, & Ender (1973) as previously described (Klein, Schams, Failing, & Hoffmann, 2003). Samples (0.1 ml) were extracted twice with 2 ml hexane. The antiserum applied was generated against 4‐pregnene‐11α‐ol‐3,20‐dione‐hemisucciate‐BSA. Intra‐ and inter‐assay coefficients of variation were 8.8 and 8.9% respectively. The limit of quantification was 0.1 ng/ml.

Measurements of E2 were performed as previously described (Hoffmann, Hoveler, Hasan, & Failing, 1992; Klein et al., 2003). Plasma samples (0.25 ml) were extracted twice with 2.5 ml toluene. The antiserum was directed against oestradiol‐17β‐6‐carboxymethyloxim‐BSA. Intra‐ and inter‐assay coefficients were 7.1 and 17.6% respectively. The limit of quantification was 2 pg/mL.

### 2.6 Analysis of creatinine via photometer

The creatinine (Cr) concentration was measured in the urine samples obtained during the OPU sessions measuring a detectable colour reaction (Jaffé‐method) using a photometer (EPAC 6140, Eppendorf, Germany).

A standard commercial kit (LT‐CR0121, LT‐SYS Labor + Technik GmbH, Berlin, Germany) was used as recommended by the manufacturer. The concentration of Cr was determined as the reference level for BAs in urine. The concentration of Cr is not influenced by nutrition and/ or protein metabolism (Kraft & Dürr, 2005). Moreover, it is subjected to a largely glomerular filtration in the kidney. Therefore, it can be used to calculate the different residence time of urine in the bladder to relativize the influence of the dilution (Bile acids [ng/mL]/ Creatinine [mg/mL] =ng BA/ mg Cr).

Blood collected during the physiological oestrus cycle was also analysed for total cholesterol (Kraft & Dürr, 2005; Leroy et al., 2004; Sanchez et al., 2014). A commercial kit (LT‐CH 0101; Labor + Technik Eberhardt Lehmann GmbH; Berlin; Germany) was used as recommended by the manufacturer.

### 2.7 Statistical analyses

Data of quantifiable BA derivates in serum and FF, obtained within the OPU sessions, were investigated for calculation of a relation of the BA concentration in both body fluids. Hence, for quantifiable BA subspecies concentration in serum and FF pairwise linear correlation analysis (Pearson correlation coefficient) was performed. Data from all cattle used for OPU were pooled for each BA subspecies of serum. Also, FF data were pooled from all classes of follicles and cattle for each BA subspecies.

Moreover, the linear correlation (Pearson correlation coefficient) was tested for the concentration of total and unesterified cholesterol in serum, collected within a physiological oestrus cycle. Data from all cattle used for serum collection were pooled for free and total cholesterol.

A *p* value less than 0.05 was considered to be significant. All statistical analyses were performed with SigmaStat (version 3.5, Systat Software GmbH, Erkrath, Germany).

## 3 RESULTS

### 3.1 Quantification of follicular BA subspecies obtained during the OPU sessions

**Figure 1:**
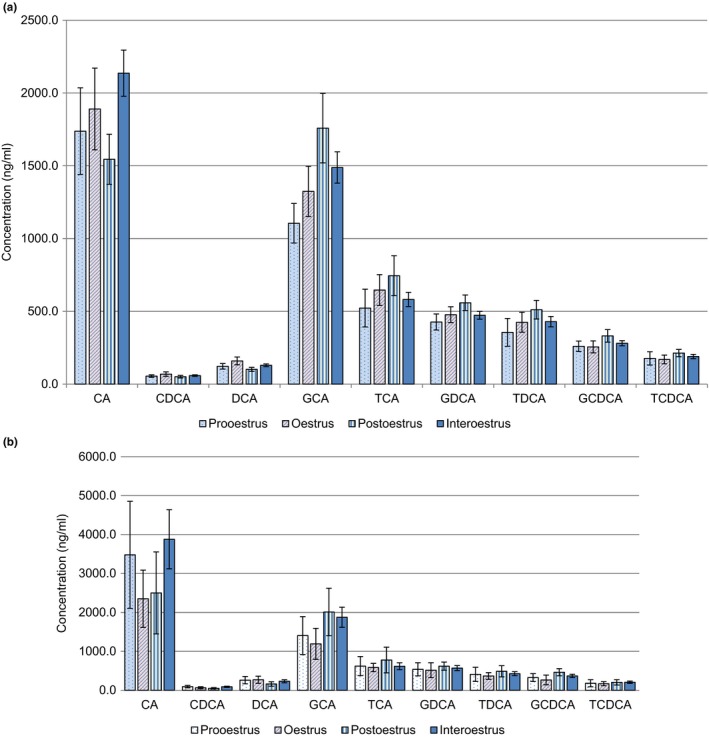
Concentration (ng/ml) of BAs (CA: cholic acid, CDCA: chenodeoxycholic acid, DCA: deoxycholic acid, GCA: glycocholic acid, TCA: taurocholic acid, GDCA: glycodeoxycholic acid, TDCA: taurodeoxycholic acid, GCDCA: glycochenodeoxycholic acid, TCDCA: taurochenodeoxycholic acid) in different fluids. The results are shown as mean ± *SEM*. (a) BA derivatives in follicular fluid obtained via OPU. (b) BA derivatives in serum obtained during OPU sessions

CA and GCA are the dominate BAs in FF irrespective of the oestrus cycle stage. Moreover, CDCA was the BA in lowest concentration in FF during the whole oestrus cycle. Data are shown in Table 2 and Figure 1a.

### 3.2 Quantification of BA subspecies in blood of cycling heifers

The concentrations of BAs in blood collected during two physiological oestrus cycles are shown in Table 1 and Figure S1. The BAs in highest concentrations were CA and its conjugated form GCA. The lowest concentrations were measured for CDCA, LCA, GLCA and TLCA.

**Table 1:** Mean (min.‐max.) concentration of BA derivates in serum of heifers

	Serum (Cycle)				Serum (OPU)			
	Pro‐oestrus	Oestrus	Post‐oestrus	Interoestrus	Pro‐oestrus	Oestrus	Post‐oestrus	Interoestrus
**Cholic acid (CA) derivatives, mean % of total**	75.0%	69.1%	68.6%	61.8%	75.4%	71.5%	72.8%	77.2%
Cholic acid (CA)	3,321.8 (2,116.6–4681.6)	3,304.8 (706.0–9148.0)	2,739.6 (1,421.0–4411.2)	1,550.6 (720.1–2543.6)	3,478.7 (154.6–4979.4)	2,350.5 (446.9–4771.5)	2,499.8 (368.3–7566.1)	3,878.6 (327.7–13743.9)
Glycocholic acid (GCA)	1,219.1 (421.9–2764.0)	4,653.4 (2,779.1–11784.7)	2,798.5 (767.0–5109.9)	2,474.9 (455.5–5796.9)	1,404.5 (206.5–2926.6)	1,191.5 (390.9–3105.0)	2010.2 (533.2–4672.8)	1874.9 (51.0–5535.8)
Taurocholic acid (TCA)	486.9 (268.1–935.4)	2,680.3 (899.9–7719.4)	1,287.1 (305.9–2963.8)	1,436.7 (197.6–4431.8)	619.9 (98.0–1520.8)	583.4 (151.9–933.4)	776.5 (209.6–2367.9)	615.8 (36.7–1925.1)
**Chenodeoxycholic acids (CDCA) derivatives, mean % of total**	7.1%	9.0%	9.2%	12.1%	8.1%	8.6%	9.8%	8.0%
Chenodeoxycholic acids (CDCA)	64.0 (41.7–76.8)	59.6 (22.5–130.9)	58.5 (26.5–96.6)	49.5 (31.8–76.4)	91.5 (97–201.1)	63.5 (14.4–187.7)	50.1 (12.0–119.3)	88.8 (10.5–332.3)
Glycochenodeoxycholic acid (GCDCA)	230.1 (99.1–468.5)	747.7 (321.2–1402.2)	496.8 (154.2–869.1)	569.0 (231.8–1348.4)	324.9 (27.8–722.5)	263.9 (14.7–830.2)	461.8 (180.5–681.7)	367.7 (19.3–854.6)
Taurochenodeoxycholic acid (TCDCA)	179.8 (59.5–375.8)	584.2 (191.2–1266.6)	362.5 (47.9–760.6)	449.7 (83.5–1052.8)	178.5 (13.9–603.9)	167.5 (34.5–352.1)	201.9 (30.6–474.6)	205.1 (7.7–494.1)
**Deoxycholic acid (DCA) derivatives, mean % of total**	16.4%	20.6%	20.7%	24.5%	16.5%	19.9%	17.4%	14.8%
Deoxycholic acid (DCA)	223.7 (130.5–359.8)	207.9 (63.2–447.7)	180.9 (87.2–285.8)	104.7 (60.0–234.6)	259.6 (4.0–584.1)	268.9 (36.4–598.8)	160.7 (19.1–417.3)	230.6 (21.3–744.3)
Glycodeoxycholic acid (GDCA)	462.7 (206.8–1027.0)	1,458.1 (812.9–2760.7)	1,002.5 (220.5–1504.5)	930.7 (227.9–1930.7)	536.7 (102.6–1018.1)	514.5 (211.7–1447.5)	618.8 (295.0–1010.8)	568.9 (24.9–1286.9)
Taurodeoxycholic acid (TDCA)	413.2 (212.9–795.8)	1511.1 (561.5–3334.8)	872.4 (211.7–1451.6)	1,133.0 (209.1–2906.9)	407.2 (55.4–1205.6)	365.4 (85.8–658.9)	486.7 (179.6–1155.2)	425.1 (78.0–946.2)
**Lithocholic acids (LCA) derivatives, mean % of total**	1.5%	1.2%	1.5%	1.6%				
Lithocholic acids (LCA)	8.8 (7.0–15.9)	*n*.d.	*n*.d.	*n*.d.	*n*.m.	*n*.m.	*n*.m.	*n*.m.
Glycolithocholic acid (GLCA)	40.9 (29.4–62.7)	79.0 (56.2–121.3)	63.7 (23.9–94.8)	53.5 (9.9–97.5)	*n*.m.	*n*.m.	*n*.m.	*n*.m.
Taurolithocholic acid (TLCA)	48.2 (31.6–73.9)	103.6 (57.2–177.7)	86.3 (34.1–115.4)	84.3 (38.9–138.1)	*n*.m.	*n*.m.	*n*.m.	*n*.m.

Note

Cycle = samples collected during a physiological oestrus cycle; OPU = samples collected during corresponding OPU sessions; *n*.d., not detectable; *n*.m., not measured; Unit: ng/ml

The profile of the different BA subspecies in blood collected during the OPU sessions showed similar values compared to those in blood collected during the physiological oestrus cycles. The highest amounts were determined for CA and its amino acid conjugate GCA. The lowest concentrations were measured for CDCA. Results are shown in Tables 1, 2 and Figure 1b.

**Table 2:** Mean (min.‐max.) concentration of BA derivates in different body fluids of heifers collected during corresponding OPU sessions

	Serum (ng/mL)				Follicular Fluid (ng/mL)				Urine (ng BA/ mg Cr)			
	Pro‐oestrus	Oestrus	Post‐oestrus	Interoestrus	Pro‐oestrus	Oestrus	Post‐oestrus	Interoestrus	Pro‐oestrus	Oestrus	Post‐oestrus	Interoestrus
**Cholic acid (CA) derivatives, mean % of total**	75.4%	71.5%	72.6%	77.2%	70.7%	71.3%	69.6%	73.0%				
Cholic acid (CA)	3,478.7 (154.6–4979.4)	2,350.5 (446.9–4771.5)	2,499.8 (368.3–7566.1)	3,878.6 (327.7–13743.9)	1738.0 (204.1–4069.7)	1,890.5 (568.9–4317.1)	1544.7 (494.2–2926.0)	2,136.5 (120.5–6745.1)	1,259.7 (76.0–3017.8)	1617.3 (104.1–4141.4)	644.0 (82.6–1495.4)	1858.9 (79.4–6447.7)
Glycocholic acid (GCA)	1,404.5 (206.5–2926.6)	1,191.5 (390.9–3105.0)	2010.2 (533.2–4672.8)	1874.9 (51.0–5535.8)	1,105.1 (499.3–1785.5)	1,324.3 (364.8–3255.4)	1758.9 (510.9–3803.4)	1,488.4 (114.1–4763.4)	126.0 (84.2–178.3)	135.0 (77.5–236.1)	141.8 (66.6–293.3)	172.8 (70.2–447.0)
Taurocholic acid (TCA)	619.9 (98.0–1520.8)	583.4 (151.9–933.4)	776.5 (209.6–2367.9)	615.8 (36.7–1925.1)	522.4 (114.1–1568.7)	646.3 (182.8–1742.2)	744.6 (129.4–1933.3)	581.4 (17.5–1901.9)	50.6 (4.0–102.5)	56.0 (34.9–97.3)	51.4 (28.5–89.6)	54.3 (13.0–144.2)
**Chenodeoxycholic acids (CDCA) derivatives, mean % of total**	8.1%	8.6%	9.8%	8.0%	10.3%	9.1%	10.2%	9.2%				
Chenodeoxycholic acids (CDCA)	91.5 (97–201.1)	63.5 (14.4–187.7)	50.1 (12.0–119.3)	88.8 (10.5–332.3)	54.8 (9.8–118.3)	68.4 (8.2–261.0)	50.6 (17.5–163.5)	58.2 (5.5–306.6)	*n*.d.	*n*.d.	*n*.d.	*n*.d.
Glycochenodeoxycholic acid (GCDCA)	324.9 (27.8–722.5)	263.9 (14.7–830.2)	461.8 (180.5–681.7)	367.7 (19.3–854.6)	259.5 (78.9–475.5)	255.5 (64.1–644.3)	331.3 (115.9–652.7)	280.6 (7.0–489.8)	*n*.d.	*n*.d.	*n*.d.	*n*.d.
Taurochenodeoxycholic acid (TCDCA)	178.5 (13.9–603.9)	167.5 (34.5–352.1)	201.9 (30.6–474.6)	205.1 (7.7–494.1)	176.4 (33.4–548.7)	169.2 (21.0–429.4)	213.0 (17.7–357.9)	189.2 (10.5–588.4)	*n*.d.	*n*.d.	*n*.d.	*n*.d.
**Deoxycholic acid (DCA) derivatives, mean % of total**	16.5%	19.9%	17.4%	14.8%	19.0%	19.6%	20.1%	17.9%				
Deoxycholic acid (DCA)	259.6 (4.0–584.1)	268.9 (36.4–598.8)	160.7 (19.1–417.3)	230.6 (21.3–744.3)	122.2 (14.3–245.2)	159.1 (35.2–394.7)	101.3 (21.2–247.1)	129.1 (11.1–390.7)	37.7 (5.8–81.2)	38.6 (11.9–62.3)	24.2 (7.5–44.6)	37.5 (7.8–128.8)
Glycodeoxycholic acid (GDCA)	536.7 (102.6–1018.1)	514.5 (211.7–1447.5)	618.8 (295.0–1010.8)	568.9 (24.9–1286.9)	426.6 (143.1–767.3)	476.7 (183.7–950.8)	558.5 (209.8–976.8)	472.8 (47.8–982.2)	10.4 (7.1–16.5)	17.2 (7.4–30.1)	16.5 (8.0–35.3)	14.0 (5.4–35.9)
Taurodeoxycholic acid (TDCA)	407.2 (55.4–1205.6)	365.4 (85.8–658.9)	486.7 (179.6–1155.2)	425.1 (78.0–946.2)	355.3 (71.6–1073.2)	425.0 (51.81092.2)	511.1 (69.6–939.9)	429.1 (10.5–1215.8)	*n*.d.	*n*.d.	*n*.d.	*n*.d.

Abbreviation: n.d., not detectable.

### 3.3 Correlation of BA subspecies and cholesterol

**Figure 2:**
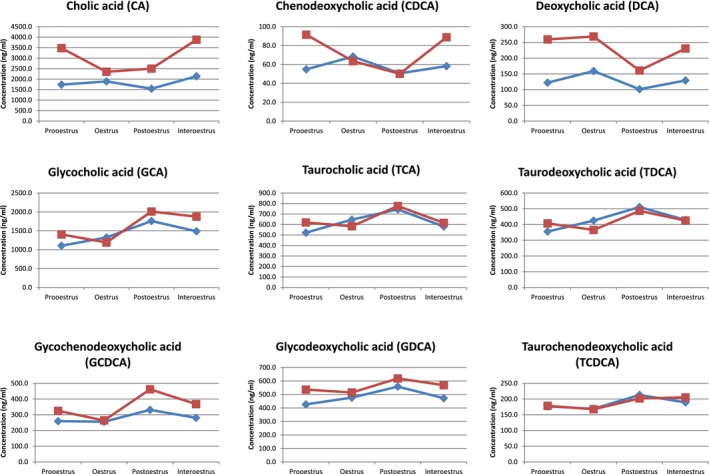
Concentration (ng/ml) of BA derivatives in follicular fluid (FF) and blood; ◊ = FF, □ = blood

Pairwise analysis for BA concentration in serum and FF yielded a strong correlation between CA in serum and CA in FF during the pro‐oestrus, oestrus and interoestrus. Also, a strong correlation was detected for GCA in serum versus GCA in FF during the oestrus and interoestrus. The correlation coefficients of the BA subspecies in serum versus FF during the different oestrus cycle stages are shown in Table 3 and Figure 2.

**Table 3:** Correlation of bile acid subspecies in serum and FF within the oestrus cycle

		Pro‐oestrus	Oestrus	Post‐oestrus	Interoestrus
**CA**	r	0.939	0.974	0.644	0.442
	*p*	.00548	.000974	.168	.0146
	*n*	6	6	6	30
**CDCA**	r	0.771	0.0425	0.997	0.541
	*p*	.229	.957	.00302	.00244
	*n*	4	4	4	29
**DCA**	r	0.866	0.846	0.961	0.434
	*p*	.0578	.0339	.00222	.0167
	*n*	5	6	6	30
**GCA**	r	0.775	0.867	0.756	0.509
	*p*	.0702	.0253	.0821	.00408
	*n*	6	6	6	30
**TCA**	r	0.839	0.791	0.879	0.554
	*p*	.0368	.0607	.0209	.00181
	*n*	6	6	6	29
**GDCA**	r	0.842	0.660	0.741	0.460
	*p*	.0356	.154	.0918	.0105
	*n*	6	6	6	30
**TDCA**	r	0.775	0.726	0.713	0.455
	*p*	.0700	.102	.112	.0151
	*n*	6	6	6	28
**GCDCA**	r	0.947	0.825	0.690	0.332
	*p*	.00417	.0431	.129	.0843
	*n*	6	6	6	28
**TCDCA**	r	0.850	0.764	0.639	0.403
	*p*	.0681	.0772	.172	.0333
	*n*	5	6	6	28

Abbreviations: *n*, number of repetitions; *p* significance level, *p* ≤ .05; *r*, correlation coefficient.

Pairwise analysis of free versus total cholesterol concentrations in serum during the physiological oestrus cycle resulted in a significant correlation, irrespective of the cycle stage (Table 4).

**Table 4:** Correlation of total and free cholesterol in serum

	Pro‐oestrus	Oestrus	Post‐oestrus	Interoestrus
r	0.987	0.957	0.976	0.991
*p*	.00167	.00272	.000831	.0000132
*n*	5	6	6	7

Abbreviations: *r*, correlation coefficient; *p* significance level, *p* ≤ .05; *n*, number of repetitions.

### 3.4 Quantification of BA subspecies in urine of cycling heifers

In urine, CA showed the highest concentration of all BAs, whereas GDCA was determined in the lowest concentrations. CDCA, TDCA, GCDCA and TCDCA were not detectable. The concentrations of BAs in urine were related to urine Cr concentration to eliminate variations in urine density. Also in relation to the Cr concentration, the BA derivatives show the same proportionality. Still CA is the BA with the highest concentration and GDCA with the lowest one. The results are shown in Table 2 and Figure S2.

## 4 DISCUSSION

BAs are usually known to be involved in physiological processes like solubilization of dietary lipids. But recently published data suggested a relation between BAs and reproduction in humans (Nagy et al., 2015; Smith et al., 2009) and cattle (Sanchez et al., 2014; Sanchez‐Guijo, Blaschka, et al., 2016), which opened a new field of research.

The role of BA within folliculogenesis and their origin are still unknown. On the one hand they could diffuse from blood to FF through the follicular wall. In this case it should be assumed that the concentration in blood and FF should be nearly identical. Nonetheless, it is known that follicular development is associated with a steady increase in size and hence fluid accumulation in the follicle. This would suggest an increasing dilution of the BA subspecies with the increase in follicle size. Data from this study do not support this hypothesis. No statistical differences were detected in BA concentrations throughout the oestrus cycle (Table 1). A possible explanation could be a continuous diffusion into the follicle.

On the other hand, BA could be actively synthesized by the follicular cells as postulated by Smith et al. (2009). Therefore, the content in FF compared to blood would be expected to be higher. Smith et al. (2009) detected higher concentrations of BAs in human FF than in serum collected 30 min after the FF collection. In addition, Nagy et al. (2015) reported higher values of BAs in FF than in serum. In the present study concentrations in blood either collected during the physiological oestrus cycle or the OPU sessions compared to those in the FF were similar or slightly higher (Figure 2). Therefore, we speculate that the BAs in bovine FF were mainly diffused through the cell layers of the follicle wall. The significant positive correlation of bile acid derivatives in blood and FF reinforces this hypothesis (see section 3.3). Moreover, this might be confirmed by the fact that it was possible to quantify DCA, a secondary BA. Secondary BAs are generated by intestinal bacteria, and therefore their presence in FF would not be possible if the only source of BAs was ‘de novo’ synthesis. Hence, this supports a diffusion process of BAs from blood to FF in cattle. If the BAs were accumulated in the follicle alone via diffusion processes, the concentration would have to decrease with increasing size of the follicle due to a dilution related to the increasing volume of the FF. Further on, a decreasing BA synthesis of follicular cells could be assumed. Sanchez et al. (2014) determined that the amount of total BAs in FF of the dominant follicle were lower than the plasma concentrations. Furthermore, they observed threefold higher concentrations of total BAs in FF of dominant follicles and plasma originating from lactating cows than in those from heifers. In the current study, samples were collected only from heifers. A possible effect of the lactation respectively the suggested ‘metabolic stress’ (Sanchez et al., 2014) could not be confirmed. Both Sanchez et al. (2014) and Smith et al. (2009) measured the total BA concentration with a colorimetric kit. Hence, the present study determined a more detailed spectrum of the BA subspecies in serum and FF in correlation to the oestrus cycle. Moreover, the difference in the amount of BA in serum and FF could also be studied.

BAs seem to be always present in FF and might have an impact for the oocytes' and resulting embryos' quality (Nagy et al., 2015). However, only little is known about the distribution pattern of BAs in FF, blood and urine depending on the oestrus cycle. Therefore, in the present study profiles of BA subspecies were determined in FF, blood and urine. In a previous study, (Sanchez‐Guijo, Blaschka, et al., 2016) CA and GCA, the conjugated form as the predominant BAs, could determine in FF collected form slaughterhouse‐derived ovaries at one oestrus cycle stage, the interoestrus. Those results are confirmed by the data obtained in this study. In FF referring to the entire oestrus cycle stage collected during the OPU sessions, CA and its amino acid conjugate (GCA) were also the major BAs. In contrast, in humans, CDCA and its conjugated forms were the predominant BA in FF (Nagy et al., (2015)).

This species‐specificity also seems to be present in blood. In humans, CA and CDCA are the primary BAs and LCA and DCA the major secondary BAs whereas in rodents and bears muricholic acid (MCA) and ursodeoxycholic acid (UDCA) are the primary BAs (Bathena, Mukherjee, Olivera, & Alnouti, 2013; Huang, Bathena, Csanaky, & Alnouti, 2011; Washizu, Tomoda, & Kaneko, 1991). CDCA has been detected as the major BA in human serum. In comparison, as already reported, (Washizu et al., 1991) CA and GCA are the main BAs in bovine serum. This might indicate an affinity for conjugation with glycine in bovine serum. CA and its amino acid conjugated form (GCA) were the predominant BAs in serum of heifers irrespective of the oestrus cycle stage (Table 1, Figure 1). In contrast, CDCA was detected with the lowest concentrations in bovine serum during the entire cycle.

The precursor of BAs and steroids is cholesterol (Kraft & Dürr, 2005). In blood, the main fraction is ligated to unsaturated fatty acids and transported with lipoproteins, because of its low water solubility. The metabolism is regulated by the liver and the concentration is correlated with the feed uptake and milk yield in cattle (Kraft and Dürr, 2005; Thomas, 2007). The present study determined a strong correlation between the unesterified cholesterol and the total cholesterol themselves.

BAs can also be cytotoxic and exhibit pathological effects if they accumulate in high concentrations. Therefore, in humans, the main pathway for detoxification and elimination of BAs is the sulfation. Bathena et al. (2013) reported a > 89% of BA sulfation in human urine. Nevertheless, in mice the elimination by means of sulfation followed by urinary excretion seems to be a minor pathway (Huang et al., 2011). The present study demonstrated an excretion of different BA subspecies, especially of the major derivatives in blood and FF, in urine from heifers. These might suggest an extraction without sulfation.

## 5 CONCLUSION

This study reports the simultaneous and direct quantification of BAs in different body fluids such as serum, FF and urine during the oestrus cycle of cattle. Nevertheless, as data were obtained from four animals throughout two complete oestrus cycles, further studies will be needed to clarify the origin and the possibility of BAs synthesis via follicular cells. The knowledge about the impact of BAs on oocyte growth and maturation as well as on subsequent embryo development still needs to be determined in detail.

## 6 ETHICS STATEMENT

The authors confirm that the ethical policies of the journal, as noted on the journal's author guidelines page, have been adhered to and the appropriate ethical review committee approval has been received. The Directive 2010/63/EU for the protection of animals used for scientific purposes were followed.

## CONFLICT OF INTEREST

We confirm that the manuscript has been read and approved by all named authors and that there are no other persons who satisfied the criteria for authorship but are not listed. We further confirm that the order of authors listed in the manuscript has been approved by all authors.
