# Supplementary material for: Profile of bile acid subspecies is similar in blood and follicular fluid of cattle
Source: Vet Med Sci. 2019 Nov 11;6(2):167–76. doi: 10.1002/vms3.217 (PMC7196682; doi:10.1002/vms3.217)
Supplement: Supplementary file 1 [file VMS3-6-167-s001.pdf]

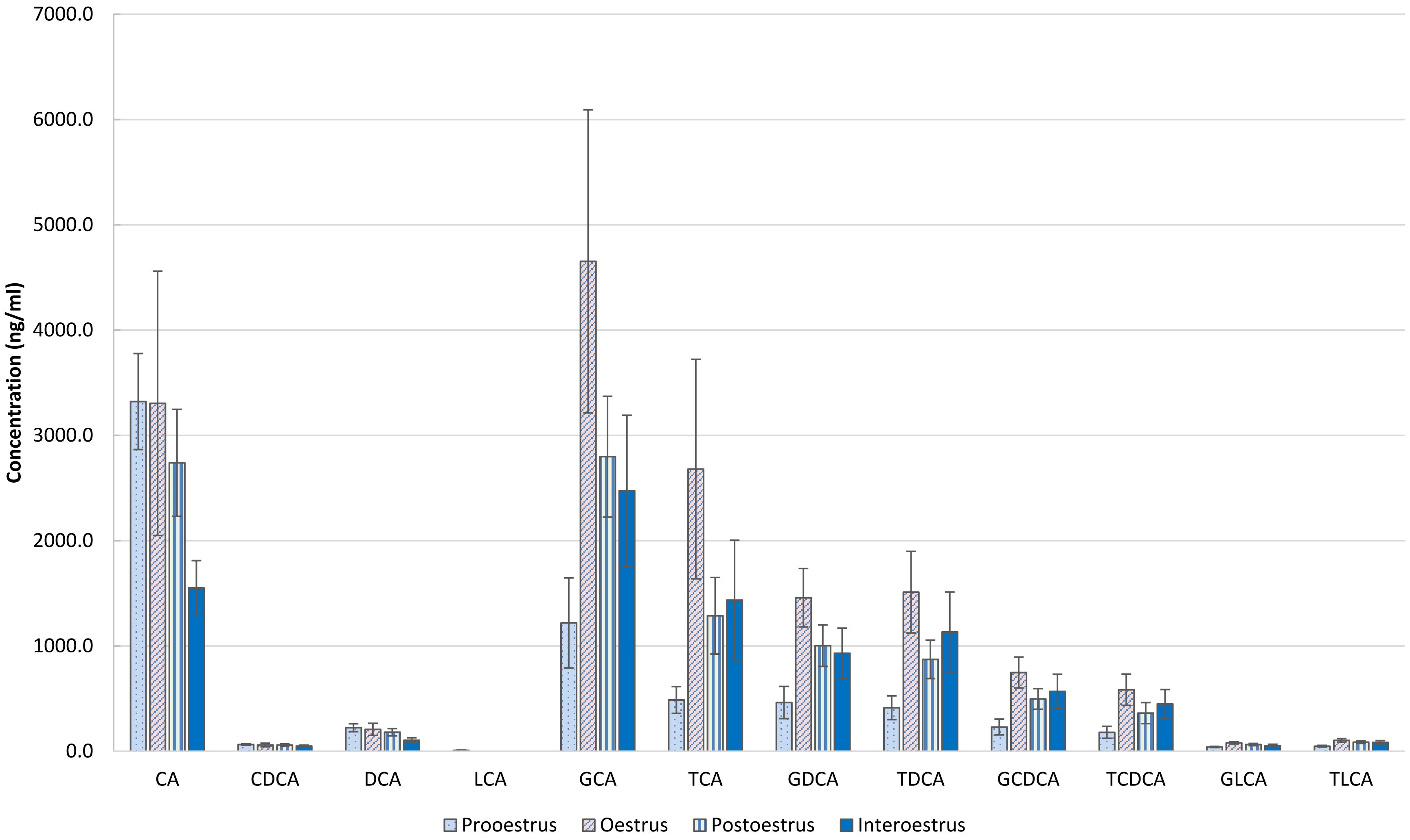

Supplemental Figure 1:
Concentration (ng/ml) of BA derivatives in serum during the physiological oestrus cycle. The results are shown as mean ± SEM.
